# Intratumoral HLA-DR^−^/CD33^+^/CD11b^+^ Myeloid-Derived Suppressor Cells Predict Response to Neoadjuvant Chemoradiotherapy in Locally Advanced Rectal Cancer

**DOI:** 10.3389/fonc.2020.01375

**Published:** 2020-08-12

**Authors:** Erez Hasnis, Aviva Dahan, Wissam Khoury, Daniel Duek, Yael Fisher, Alex Beny, Yuval Shaked, Yehuda Chowers, Elizabeth E. Half

**Affiliations:** ^1^Department of Gastroenterology, Rambam HealthCare Campus, Haifa, Israel; ^2^Cancer Center, Sanford-Burnham-Prebys Medical Discovery Institute, San Diego, CA, United States; ^3^Department of Colorectal Surgery, Rambam HealthCare Campus, Haifa, Israel; ^4^Department of Pathology, Rambam HealthCare Campus, Haifa, Israel; ^5^Department of Oncology, Rambam HealthCare Campus, Haifa, Israel; ^6^Department of Cell Biology and Cancer Science, Rappaport Faculty of Medicine, Technion – Israel Institute of Technology, Haifa, Israel

**Keywords:** myeloid-derived suppressor cells, neoadjuvant chemoradiotherapy, locally advanced rectal cancer, response to therapy, intratumoral MDSCs

## Abstract

Capecitabine-based neoadjuvant chemoradiation therapy (nCRT) is currently the mainstay of treatment for locally advanced rectal cancer (LARC), prior to surgical tumor removal. While response to this treatment is partial, it carries significant risk of side effects. As of today, there is no accepted model to predict tumor response, and allow for patient stratification. The level of circulating Myeloid-derived suppressor cells (MDSCs), a subpopulation of early myeloid cells (EMCs), has been shown to correlate with prognosis and response to therapy in advanced colon cancer, but their role in LARC is not clear. We sought to study the effect of intratumoral and circulating levels of different EMCs subpopulations including MDSCs on response to nCRT. We analyzed tumor, normal mucosa, and peripheral blood samples from 25 LARC patients for their different EMCs subpopulation before and after nCRT, and correlated them with degree of pathologic response, as determined postoperatively. In addition, we compared LARC patient to 10 healthy donors and 6 metastatic patients. CD33^+^HLA-DR^−^CD16^−^CD11b^+^EMCs in the circulation of LARC patients were found to inhibit T-cell activation. Furthermore, elevated levels of CD33^+^HLA-DR^−^ myeloid cells were found in the tumor relative to normal mucosa, but not in the circulation when compared to healthy subjects. Moreover, intratumoral, but not circulating levels of MDSCs correlated with clinical stage and response to therapy in patients treated with nCRT, with high levels of MDSCs significantly predicting poor response to nCRT. Importantly, therapy by itself, had significant differential effects on MDSC levels, leading to increased circulating MDSCs, concomitantly with decreasing intratumoral MDSCs. Our results suggest that high levels of intratumoral, but not circulating MDSCs may confer drug resistance due to immunomodulatory effects, and serve as a biomarker for patient stratification and decision-making prior to nCRT.

## Introduction

Colorectal cancer (CRC) is one of the most prevalent malignancies, with 140,250 new cases in 2018 in the US (1). A quarter of these tumors are confined to the distal 12 centimeters of the colon, and represents the subgroup of rectal adenocarcinoma (RAC). Although mostly similar to the more proximal colon cancer in biological behavior and metastasis, the local recurrence of RAC is much higher. Currently, neoadjuvat chemoradiotherapy (nCRT), such as 5-fluorouracil (5-FU) based-chemotherapy and radiation is the cornerstone of therapy for locally advanced rectal cancer (LARC, stage II and III) (2–4). While pathologic complete response (pCR), defined as the total microscopic clearance of tumor cells in the rectal wall, is the therapeutic goal, (5) it is only achieved in ~10% of individuals. While any response to this treatment modality is achieved in only about 50% of these patients, it carries significant side effects such as damage to the anal sphincters resulting in fecal incompetence (3), radiation proctitis with mucosal injury leading to chronic proctitis anemia and rectal ulcers (4), and neural insult with male impotence (6) in addition to increase in surgical complication rates. Taken together, the standard capecitabine-based nCRT is only partially effective yet carries significant side effects, and some degree of residual disease after therapy usually persist. Several studies have focused on identifying patient who will benefit from nCRT while avoiding therapy and permitting early surgical treatment from those who will not (7–10). Currently, although intense research into genetic (7), molecular (8), and clinical (9, 10) components of the disease, there is no effective method to predict which patients will respond to the treatment. For example, patients who will be predicted as non-responders, could go directly to surgery, while patients with prediction of pCR could be treated with nCRT and then potentially followed by a “wait and see” approach. Thus, a predictive biomarker for patient selection for chemoradiation pretreatment is desperately needed.

A growing body of evidence suggests a role for early myeloid cells (EMC) in the pathogenesis and progression of many types of cancer (11). In some malignancies, these myeloid precursor cells are released immaturely from the bone marrow to the circulation by mechanisms which are not completely understood. Some of the circulating EMCs, and in several clinical situations also tumor infiltrating EMC populations, are able to suppress both innate and adaptive immune responses (12). They do so, both by the production of nitric oxide (NO), reactive oxygen and nitrogen species (RONS), and by a direct inhibition of tumor-infiltrating lymphocytes (12). Therefore, they are also termed myeloid-derived suppressive cells (MDSCs) (13). MDSCs have been extensively investigated both in human and mice. In mice, MDSCs are more abundant in the peripheral circulation relative to humans, and are identified as GR1^+^/CD11b^+^ cells that can be further subdivided into two main subsets with different phenotypic and biologic properties. The monocytic MDSCs (Mo-MDSC) are characterized as CD11b^+^/Ly6G^−^/Ly6C^high^, and the granulocytic-like MDSCs (GMDSC) express CD11b^+^/Ly6G^+^/Ly6C^low^ (14). In human, however, MDSCs are a much more heterogenous population by means of cell surface markers and immunosuppressive activity. They lack the Gr-1 antigen and they seem to be less abundant in the circulation. Within the entire immature leucocytes CD33^+^ population, the CD14^+^ MDSCs share characteristics similar to the murine monocytic MDSCs (15), whereas the CD14^−^/CD66b^+^ MDSCs resemble the murine granulocytic subtype (16). Other markers of myeloid maturity, such as CD16, may further divide the MDSCs populations into smaller subpopulations, each of which with its distinct immunosuppressive effect (17).

Increased levels of MDSCs have been shown in several types of cancer, including CRC (18–20). Their numbers correlate with lack of response to therapies including chemotherapies such as 5-FU, irinotecan (CPT11) (21) and radiotherapy (22). In addition, MDSCs, have been shown to contribute to angiogenesis (23), and to contribute to the resistance of tumors to antiangiogenic drugs (24). Thus, they play a major tumor-promoting role in the tumor microenvironment. In CRC, studies have shown a correlation between high MDSC levels and chemo-resistance but only for advanced metastatic disease, and only peripherally circulating MDSCs population were studied (21). Yet, in local disease, the circulating levels of MDSCs may not be applicable, thus requiring additional studies and correlations between tumor-infiltrated MDSCs and stage of disease.

nCRT protocol for locally advanced rectal cancer include daily dosing of capecitabine (5-FU prodrug) for 6 weeks and radiotherapy for 5 weeks. It has been shown that the levels of circulating MDSCs are affected by 5-FU, that exerts direct inhibitory and pro-apoptotic effect on MDSCs (21). Interestingly, as capecitabine is administered continuously on a daily basis, it acts as a metronomic chemotherapy regimen (25). Indeed, we have previously demonstrated that metronomic dosing of chemotherapy, such as gemcitabine, may also exert anti-MDSCs effect (26). This suppressive effect of MDSCs, however, was not found when the drug was administered in high pulses of maximal tolerated dose protocol. In such cases, it was shown that MDSCs levels are rebound in the circulating and further colonize and accumulate in the treated tumors (26).

In this study, we prospectively evaluated the ability of circulating and intratumoral CD33^+^/HLA-DR^−^/CD11b^+^ EMCs populations to serve as predictive biomarkers for response to capecitabine-based nCRT in patients with locally advanced (stage II and III) patients. We found that relatively increased levels of the CD11b^+^/CD16^−^ subpopulation, within the tumor but not in the circulation, predict no pCR. These results suggest the use of these cells as biomarkers for response to nCRT, and perhaps as a cellular therapeutic target in patient exhibiting high MDSC content.

## Results

### Patients, Study Design, and Grading of Response to nCRT

**Figure 1:**
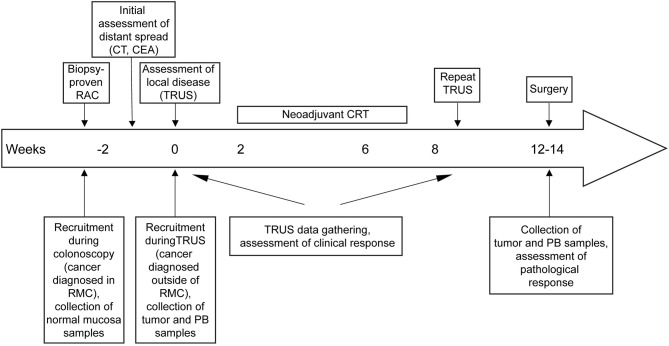
Detailed schematic model and time course of the clinical study.

Total of 33 patients were screened for inclusion in this study between October 2016 and November 2017, upon diagnosis of rectal cancer. Reason for exclusion of 8 patients are listed in Supplemental Figure 1A. Eventually, we analyzed whole blood and rectal tumor from 25 patients with stage II/III LARC amenable to nCRT (Table 1), before and after nCRT. Of those, 14 patients had undergone diagnostic colonoscopy in our medical center, and for those patients a biopsy of normal rectal mucosa, adjacent to the tumor was also obtained. Eleven of the 25 patients were referred after colonoscopy was done elsewhere and only tumor biopsies were obtained during TRUS. After determining tumor local stage by TRUS (week 0, Supplemental Figure 1B), patients were then treated with local radiation and capecitabine followed by surgical tumor resection on week 9 or 10 according to the standard treatment protocol (Figure 1). Of the 25 patients treated with nCRT, 11 patients responded to nCRT (TRG 3, no tumor cells; TRG 2 dominant fibrosis with few tumor cells 2/3). No demographic characteristics were found to be associated with lack of response to nCRT in LARC patients (Table 2). For a selected group of 15 patients, mutation analysis was available (Table 3). K-Ras mutation was found only in the non-responders group, however, sample size was too small to determine significance.

**Table 1:** Demographic details of LARC patients, metastatic patients and healthy individulas.

**Details of LARC patients, metastatic patients and HDs**	**Numbers (%)**
**LARC patients (*****n =*** **25)**		
Median age	64
**Gender**	
Male	18 (72%)
Female	7 (28%)
**Metastatic patients (*****n =*** **6)**	
Median age	59
**Gender**	
Male	2 (33.3%)
Female	4 (66.7%)
**HDs (*****n =*** **10)**	
Median age	60.5
**Gender**	
Male	7 (70%)
Female	3 (30%)

**Table 2:** Baseline clinical characteristics of LARC patients.

	**Overall**	**Post-op response**		***p*-value**
	**(*n =* 25)**	**assessment (*****n =*** **25)**		
		**Responders**	**Non-responders**	
		**(*n =* 11)**	**(*n =* 14)**	
Female gender (%)	7 (28%)	3 (27.3%)	4 (28.6%)	0.94277
**Age (ys)**				
20–29	2 (8%)	1 (9.1%)	1 (7.1%)	
30–39				
40–49	3 (12%)	2 (18.2%)	1 (7.1%)	0.07771
50–59	4 (16%)		4 (28.6%)	
60–69	7 (28%)	4 (36.4%)	3 (21.4%)	
70–79	9 (36%)	4 (36.4%)	5 (35.7%)	
**Pathology**				
Well differentiated	11 (44%)	6 (54.5%)	5 (35.7%)	
Moderately differentiated	13 (52%)	5 (45.5%)	8 (57.1%)	0.72865
Poorly differentiated	1 (4%)		1 (7.1%)	
**T Stage**				
T1				
T2	4 (16%)	3 (27.3%)	1 (7.1%)	0.10359
T3	21 (84%)	10 (90.9%)	11 (78.6%)	
T4				
**N stage**				
N0	8 (32%)	4 (36.4%)	4 (28 6%)	
N1	17 (68%)	7 (28%)	10 (71.4%)	0.67844
N2				
**Distance from anus (cm)**				
0–4	6 (24%)	2 (18.2%)	4 (28.6%)	
4–8	11 (44%)	4 (36.4%)	7 (50%)	0.43854
8–12	8 (32%)	5 (45.5%)	3 (21.4%)	
**Intratumoral EMC subpopulations relative densities (average, % of tumor) cells)**				
CD16–/CD11b–	1.38	0.90	1.85	0.16722
CD16–/CD11b+	1.35	0.63	2.06	0.02016
CD16+/ CD11b+	1.18	1.54	0.82	0.22759

**Table 3:** Specific somatic mutations in LARC patients.

	**Overall**	**Post-op response**	
	**(*n =* 15)**	**assessment (*****n =*** **15)**	
		**Responders**	**Non-responders**
		**(*n =* 7)**	**(*n =* 8)**
**K-RAS**			
G2A	2 (8%)	0 (0%)	2 (25%)
G12C	1 (6.7%)	0 (0%)	1 (12.5%)
G12S	1 (6.7%)	0 (0%)	1 (12.5%)
WT	11 (73.3%)	7 (100%)	4 (50%)
**N-RAS**			
WT	15 (100%)	7 (100%0	8 (100%)
**BRAF**			
WT	15 (100%)	7 (100%)	8 (100%)
**PIK3CA**			
WT	15 (100%)	7 (100%)	8 (100%)

*WT, wild type*.

### Elevated Levels of CD33^+^HLA-DR^−^ Myeloid Cells in the Different Compartments in Patient With Rectal Carcinoma

**Figure 2:**
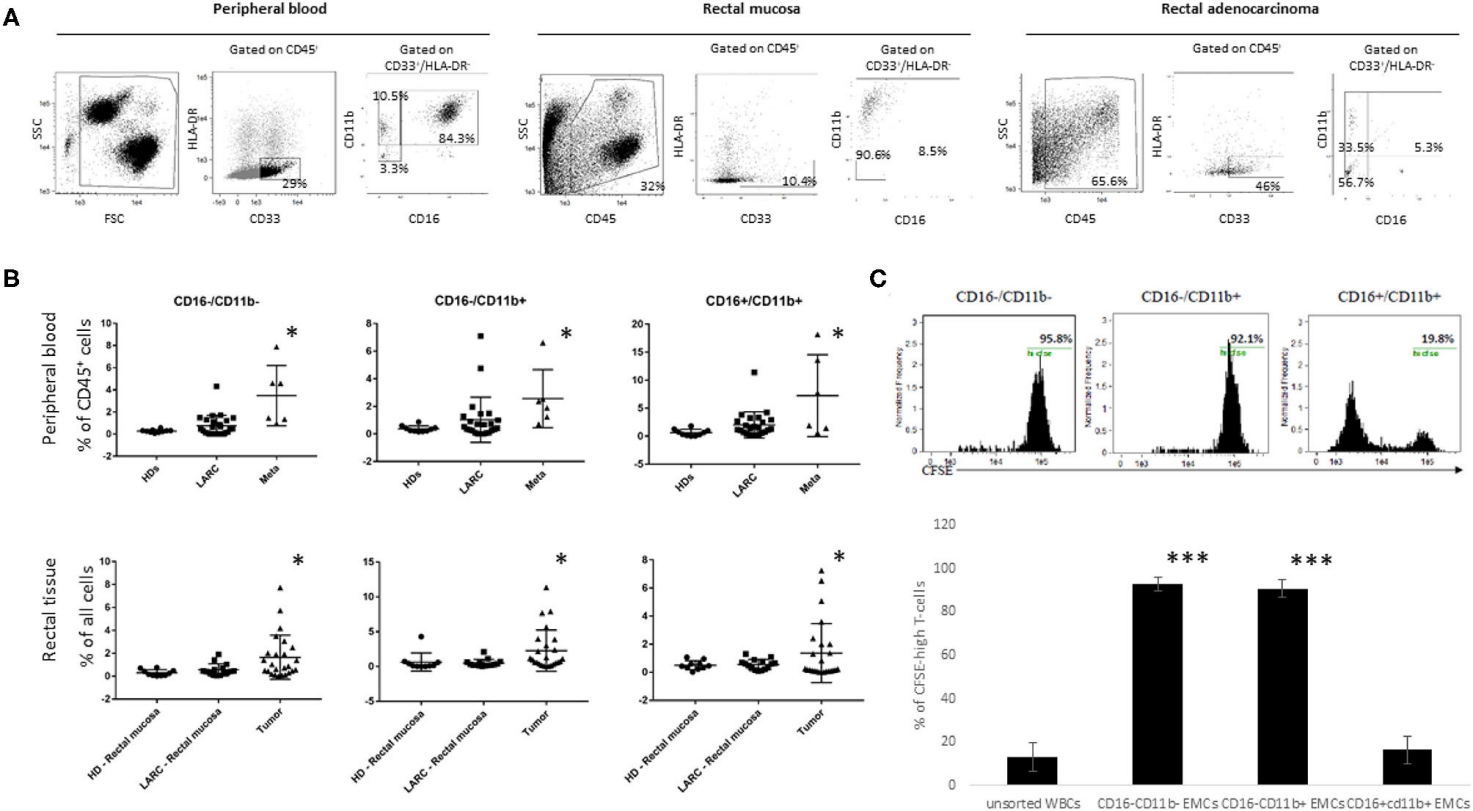
CD16^−^CD11b^−/+^EMCs levels are increased in tumor but not peripheral blood of LARC patients, and bear immunosuppressive capabilities. The different EMCs subpopulations are presented by the percentages of HLA-DR^−^CD33^+^CD16^−^CD11b^−/+^ of the entire peripheral blood or tissue population. **(A)** representative pictures of the three different EMCs populations (CD16^−^CD11b^−^, CD16^−^CD11b^+^, CD16^+^CD11b^+^), gated from CD33^+^HLA-DR^−^ population from peripheral blood, Normal mucosa, and rectal tumor of LARC patients. **(B)** flow cytometry analysis of the three different EMCs populations mentioned above in the peripheral blood (upper line) of HD (*n* = 10), LARC patients (*n* = 25) and metastatic patients (*n* = 6), and in rectal tissue (lower line) of HD (*n* = 10), rectal mucosa and rectal tumor in LARC patients (*n* = 25), *P < 0.05 **(C)** Flow cytometry analysis of CFSE content in peripheral blood T-cells directly exposed to the different subpopulations of EMCs (Upper row—representative pictures. *N* = 5 tumors, 10 healthy lymphocyte donors, ***P < 0.005).

We first assessed whether CD33^+^/HLA-DR^−^/EMCs and its subpopulations are enriched in blood samples and tumor tissue of cancer patients compared to healthy donors. We also assessed if these myeloid populations exhibit suppressive phenotype, as previously reported for CRC (21). In this study, all CD33^+^/HLA-DR^−^ cells were considered to represent EMC populations. We focused, however, on CD11b^−^/CD16^−^ which are considered to represent the most immature population of EMCs, in addition to CD11b^+^/CD16^−^ and CD11b^+^/CD16^+^ MDSCs which are considered to be EMCs in more advanced stages of maturity. Analysis of blood samples taken before the first nCRT showed that the percentage of all 3 subpopulation were not significantly higher for LARC patients when compared with HD (0.44 ± 0.29 vs. 0.21 ± 0.06 for CD16^−^/CD11b^−^, 1.14 ± 0.57 vs. 0.37 ± 0.09 for CD16^−^/CD11b^+^, 1.8 ± 0.49 vs. 0.94 ± 0.24 for CD16^+^/CD11b^+^, *P* > 0.05, Figure 2B). However, when blood samples from metastatic CRC patients were examined, CD16^−^/CD11b^−^ EMCs (3.8 ± 1.24, *P* < 0.05), CD16^−^/CD11b^+^ EMCs (2.2 ± 0.86, *P* < 0.05), and CD16^+^/CD11b^+^ EMCs (7.6 ± 3.1, *P* < 0.05) were significantly increased compared to both LARC patients and HD. When normal rectal mucosa from HD or LARC patients were compared with tumor tissue, higher percentages of all 3 subtypes of EMCs were found in the rectal tumors (Figure 2B).

Next, we analyzed the suppressive abilities of the different EMC subpopulations in rectal cancer patients. For this purpose, the different EMC subpopulations were sorted out from 5 different patients, and incubated with CFSE-prestained T-lymphocytes isolated from peripheral blood of 10 healthy donors, before activation with CD3/CD28 beads to test cell proliferation. Flow cytometry analysis of CFSE content in the lymphocytes exhibited a significant decrease in proliferation of the T-cells exposed to CD16^−^CD11b^+^ EMCs, but much less than this in T-cells exposed to CD16^−^CD11b^−^ EMCs (Figure 2C). Based on these results we further focused our research on the presence of CD16^−^CD11b^+^ EMCs in the tumor microenvironment, and considered them as MDSCs.

### Circulating MDSC Levels Do Not Correlate With Clinical Stage or Response to Therapy in LARC Patients Treated With nCRT

**Figure 3:**
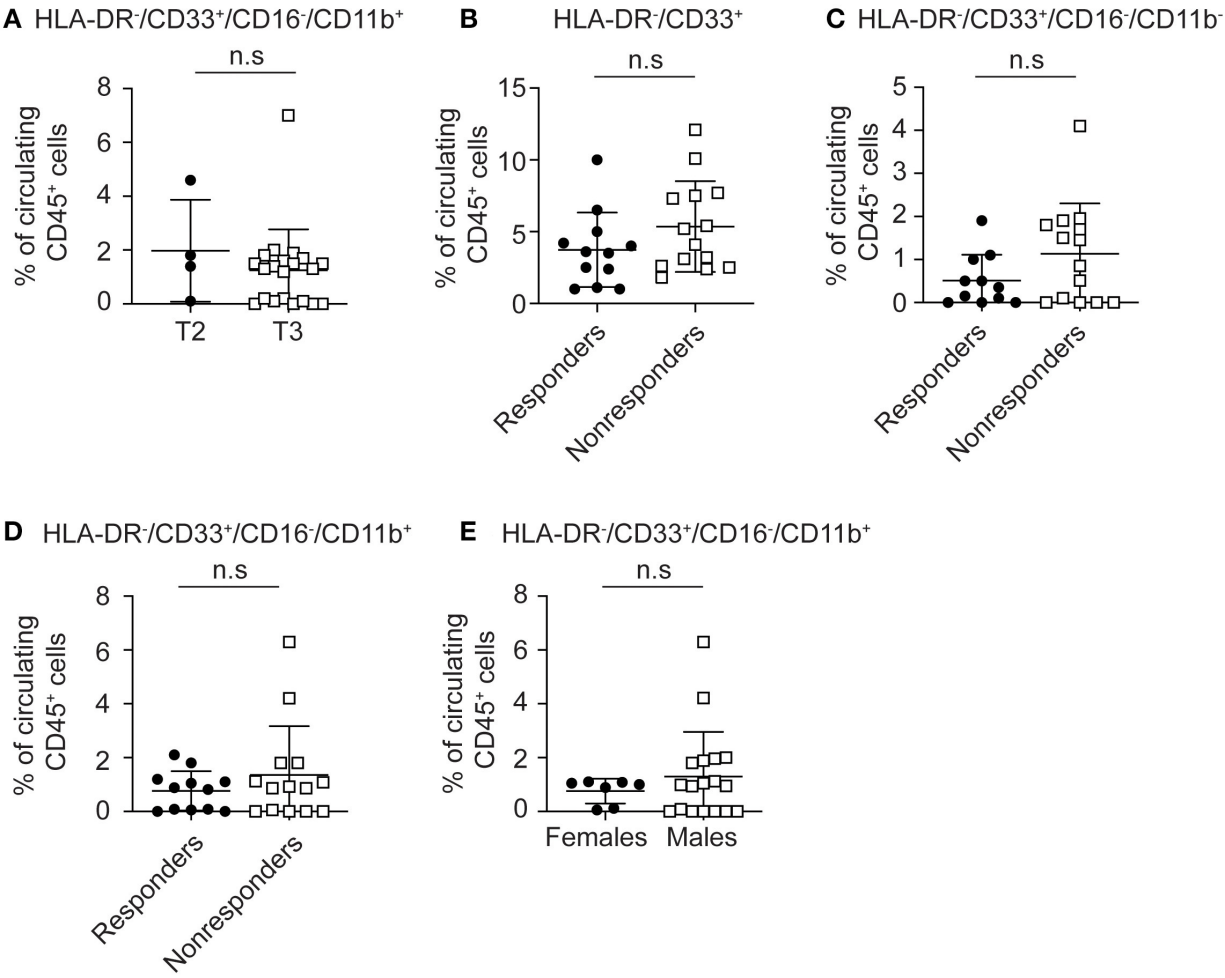
Circulatory MDSCs levels do not correlates with local tumor stage or response to nCRT. **(A)** Patients with LARC were divided into two groups (T2 and T3) according to the TNM classification by the use of TRUS, and intratumoral CD33^+^HLA-DR^−^CD16^−^CD11b^+^ MDSC levels were evaluated for each patient. **(B)** Total circulatory EMCs (CD45+HLA-DR-CD33+) were evaluated in LARC patients divided into two groups, responders and non-responders to nCRT. **(C)** Circulatory CD33^+^HLA-DR^−^ CD16^−^CD11b^−^ EMC were evaluated for each patient in the responders vs. non-responders groups. **(D)** Circulatory HLA-DR^−^CD33+CD16^−^CD11b+ EMC subpopulation levels were evaluated for each patient in the responders and non-responders groups. **(E)** Circulatory CD16^−^CD11b^+^ MDSC percentage was evaluated for each patient, with patients divided into female and male groups. n.s., not significant. The percentage of MDSCs shown here are of all CD45+ circulatory cells **(A–E)**.

Since several recent studies suggested that peripheral levels of MDSC can serve as a potential biomarker for response to therapy (22, 27, 28), we examined their levels in our study. LARC can be classified into stage T2 or T3 based on the degree of the rectal wall involvement. We determined T stage for our population using TRUS. We did not observe a significant change in circulating CD16^−^/CD11b^+^ MDSCs between T2 (1.97 ± 0.96, *n* = 4) and the T3 patients (1.03 ± 0.33, *n* = 21, *P* = 0.281; Figure 3A). We also compared circulating cells between stage II and stage III tumors. In this analysis, no significant change in circulating CD16^−^/CD11b^+^ MDSCs between stage II (1.35 ± 0.46, *n* = 9) and stage III patients (1.37 ± 0.43, *n* = 14, *P* = 0.66; Supplemental Figure 2A) was found. We then examined the ability of the different EMC subpopulations to serve as predictors of response. Total circulating early myeloid (CD33^+^/HLA-DR^−^) cells were not different in responders (3.75 ± 0.68, *n* = 11) compared to non-responders (5.42 ± 0.88, *n* = 14, *P* = 0.0928; Figure 3B). Further analysis of CD16^−^/CD11b^+^ EMCs which have been shown to serve as MDSCs, did not reveal any change in circulating levels between responders and non-responders (0.71 ± 0.19, *n* = 11 for responders, vs. 1.28 ± 0.52, *n* = 14 for non-responders, *P* = 0.312; Figure 3C). Likewise, CD16^−^/CD11b^−^ cells did not differentiate responders from non-responders (0.45 ± 0.16, *n* = 11 for responders, vs. 1.06 ± 0.3, *n* = 14 for non-responders, *P* = 0.114; Figure 3D).

Finally, we asked whether gender might affect circulating MDSC frequencies. We did not detect significant changes in circulating CD33^+^HLA-DR^−^CD16^−^CD11b^+^ MDSC levels between females and males (Figure 3E). Taken together, these results indicate that circulating MDSC levels do not correlate with disease extent or predict response to nCRT in this relatively early and localized tumor stage.

### Intratumoral MDSC Levels Correlate With Tumor Size and Predict Responsiveness in LARC Patients Treated With nCRT

**Figure 4:**
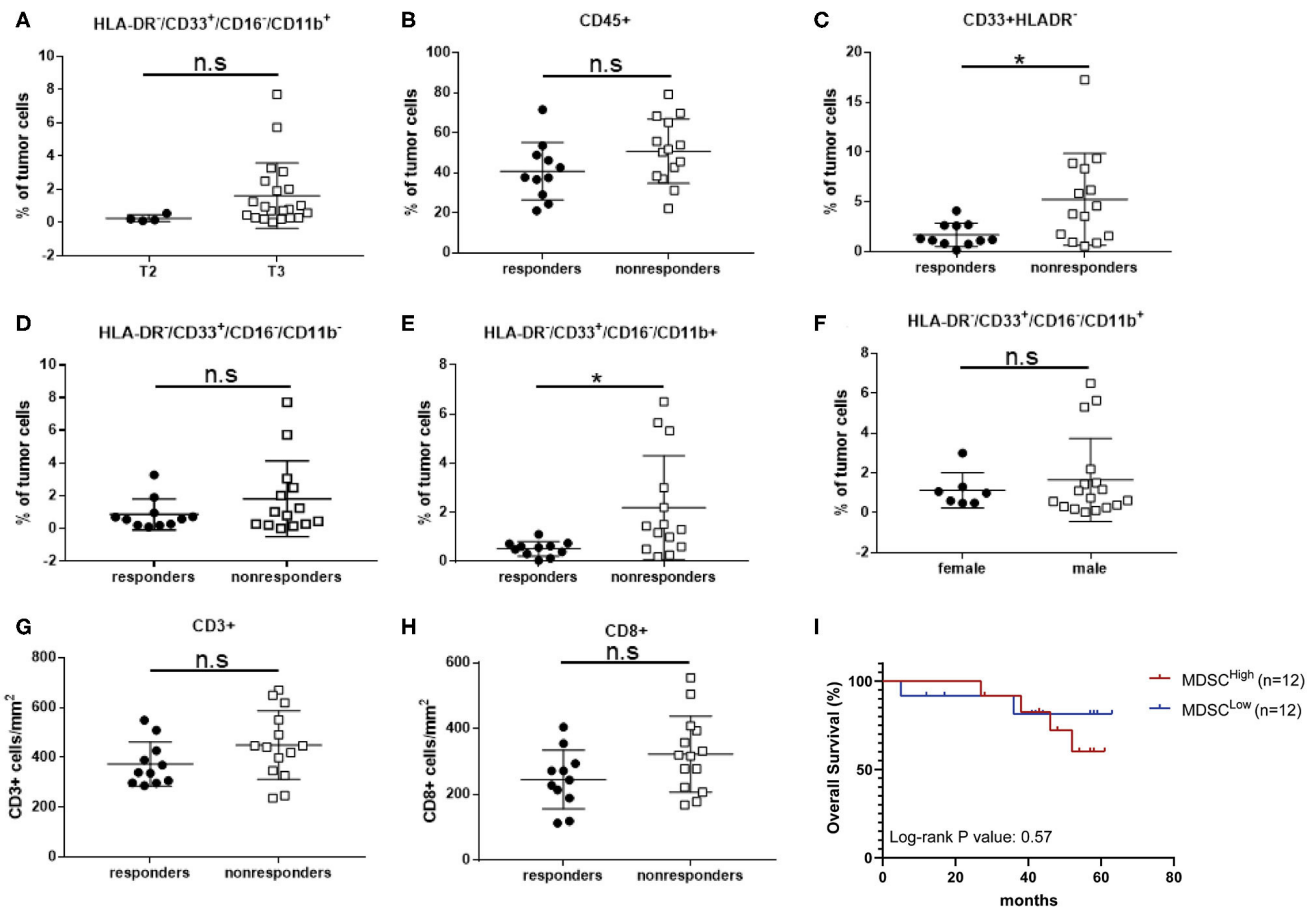
Intratumoral CD16^−^/CD11b^+^ MDSCs percentage correlates with local tumor stage and response to nCRT in LARC patients. **(A)** Patients with LARC were divided into two groups (T2 and T3) according to the TNM classification by the use of TRUS, and intratumoral CD33^+^HLA-DR^−^CD16^−^CD11b^+^ MDSC percentages were evaluated for each patient. **(B)** Total intratumoral CD45^+^ cells were evaluated in LARC patients divided into two groups, responders and non-responders to nCRT. **(C)** Intratumoral CD33^+^HLA-DR^−^ EMC percentage was evaluated for each patient in the responders vs. non-responders groups. **(D)** Intratumoral CD16^−^CD11b^−^ EMC subpopulation percentage was evaluated for each patient in the responders and non-responders groups. **(E)** Intratumoral CD16^−^CD11b^+^ MDSC percentage was evaluated for each patient in the responders and non-responders groups. **(F)** Intratumoral CD16^−^CD11b^+^ MDSC percentage was evaluated for each patient, with patients divided into female and male groups. **(G,H)** CD3^+^ **(G)** and CD8^+^ **(H)** tumor-infiltrating lymphocytes were evaluated in LARC patient, divided into responders and non-responders to nCRT. *P < 0.017 (*t*-test); n.s., not significant. The percentage of MDSCs shown here are of all tumor cells **(A–F)**.

Being localized to the rectal lumen, and many times amenable for cure by local resection, we sought to examine whether the tumor infiltrating MDSCs, rather than peripheral MDSCs, may affect tumor growth and/or drug resistance. Hence, it may serve as a biomarker for response to nCRT. Based on our results showing the immunosuppressive capability of certain CD33^+^HLA-DR^−^ MDSCs in LARC patients, and their relative intratumoral abundance, we evaluated whether their levels can correlate with clinical stage. We found that the intratumoral CD16^−^CD11b^+^ MDSC frequencies are much lower in stage T2 tumors (0.25 ± 0.1, *n* = 4) than in stage T3 tumors (1.62 ± 0.43, *n* = 21). However, since only 4 patients with T2 tumors were enrolled into this study, we were not able to achieve statistical significance for the stage difference (*P* = 0.187; Figure 4A). To consider local spread to lymph node, similar analysis was performed to compare stage II and stage III tumors. In this analysis, no significant change in tissue CD16^−^/CD11b^+^ MDSCs between stage II (1.94 ± 0.63, *n* = 9) and stage III patients (1.77 ± 0.6, *n* = 14, *P* = 0.64; Supplemental Figure 2B) was found, suggesting that in LARC, MDSCs might correlate with tumor size, but less with local spread. Because we have shown a significant increase in white cell abundance in rectal tumors compared with normal rectal mucosa (Figure 2A), we checked if white blood cell infiltration of LARC may yield change in prognosis. To this end, we analyzed the intratumoral level of the entire CD45^+^ population in responders and non-responders. High inter-sample variability was detected, with some tumors have as low as ~20% of CD45^+^ infiltrating cells, while other tumors exhibited up to 80% infiltration. However, no changes in intratumoral CD45^+^ cells between responders (40.81 ± 4.3, *n* = 11) and non-responders (50.77 ± 4.3, *n* = 14, *P* = 0.12; Figure 4B) were found.

While total leucocyte infiltration level did not affect response to therapy, we did find a significant increase in immature CD33^+^HLA-DR^−^ population tumor infiltration in non-responders (5.26 ± 1.23, *n* = 14) compared with responders (1.69 ± 0.35, *n* = 11, *P* = 0.02; Figure 4C). These results suggest that this population or any of its subset may serve as a marker for response to therapy. To further dissect this immature population, we examined the intratumoral frequencies of CD16^−^CD11b^−^ EMCs, and found that there were no significant changes in their frequencies between responders and non-responders (0.86 ± 0.286, *n* = 11 vs. 1.82 ± 0.62, *n* = 14, *P* = 0.212; Figure 4D). A significant change was observed, however, with the intratumoral levels of CD16^−^CD11b^+^ MDSCs between responders (0.52 ± 0.09, *n* = 11) and non-responders (2.19 ± 0.57, *n* = 14, *P* = 0.0167) (Figure 4E). Also, in this case, gender did not serve as a determining factor for MDSCs levels (Figure 4F).

It has been suggested that MDSCs act directly as inhibitors of cytotoxic T-cells, in part by direct contact-mediated inhibition of cytotoxic T-cells. Therefore, we analyzed intratumoral levels of CD3^+^- and CD8^+^-T cells. No differences in T-cell levels, either CD3^+^ or CD8^+^, were observed between responders and non-responders (Figures 4G,H), suggesting that in activation of MDSC does not result in depletion of tumor-infiltration lymphocytes. Since response to nCRT was identified as a predictor of overall survival (5), we analyzed the survival of patients based on their tumor MDSC content. A trend toward prolonged survival was noted in the MDSC^Low^ compared with the MDSC^High^ group, although this did not reach statistical significance (Figure 4I). Taken together, these results suggest that HLA-DR^−^/CD33^+^/CD16^−^/CD11b^+^ MDSC are a distinct and unique subpopulation, which differentiate between responder and non-responder LARC patients.

### nCRT Treatment Results in MDSC Levels Change in Blood and Tumor

**Figure 5:**
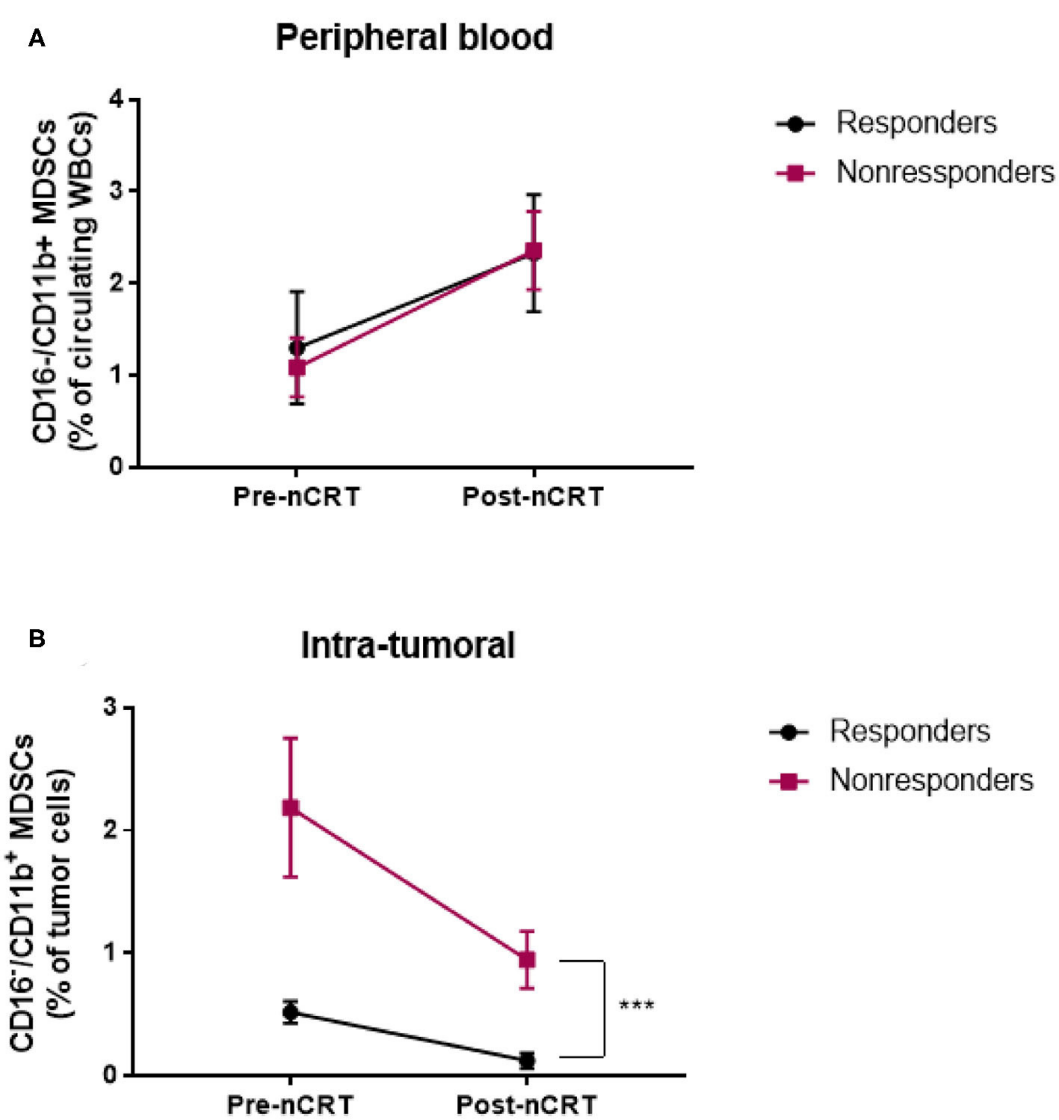
Neoadjuvant chemoradiotherapy for LARC patient is associated with changes in circulating and intratumoral MDSC levels. **(A,B)** circulating **(A)** and intratumoral **(B)** CD16^−^CD11b^+^ MDSC percentage was evaluated in LARC patients before and after nCRT. ***P < 0.001 (*t*-test); nCRT, neoadjuvant chemoradiotherapy.

Certain chemotherapeutic regimens, and among them 5-FU, have been previously shown to contribute to tumor shrinkage by inhibiting MDSC proliferation (21, 29). We therefor examined the effect of nCRT on MDSC accumulation in peripheral blood and tumor tissue of LARC patients. To this end, blood was drawn from patients on the first day of treatment, and when they next arrived for the surgical procedure, usually 6–8 weeks after completing the course of neoadjuvant therapy. Interestingly, we found an opposing effect of circulating and intratumoral MDSC levels following nCRT. Specifically, in the circulation, nCRT resulted in a significant increase in MDSCs, both in responder (1.3 ± 0.58 pre-nCRT, 2.33 ± 0.61 post-nCRT, *P* = 0.0188, *n* = 11, paired *t*-test) and non-responder (1.08 ± 0.32 pre-nCRT, 2.36 ± 0.42 post-nCRT, *P* = 0.0033, *n* = 14, paired *t*-test; Figure 5A). However, the post-nCRT circulating MDSC frequencies were not significantly different between responders and non-responders. Yet, MDSC frequencies within the tumors decreased as a result of the applied therapy. In both groups, responders and non-responders, levels of MDSCs declined by 4–5-fold (0.52 ± 0.086 pre-nCRT vs. 0.12 ± 0.06 post-nCRT for the responder group, *P* = 0.0002, *n* = 11; 2.2 ± 0.95 pre-nCRT vs. 0.57 ± 0.23 post-nCRT for the non-responder group, *P* = 0.0057, *n* = 11, paired *t*-test; Figure 5B). It is noteworthy, that in the case of complete response, MDSCs were quantified in the scar tissue. The same as for pre-nCRT levels of intratumoral MDSCs, the post-nCRT levels were also significantly higher in the non-responder group, suggesting that the mechanism underlying a decline in MDSC levels is probably similar in both groups.

## Discussion

As research continue to expand, our understanding of MDSC biology and especially their role in cancer has become increasingly complex. In addition to their role in suppressing immune response, they directly support tumor growth (30) and metastasis (31). In the present study, we evaluated the clinical relevance of circulating and intratumoral CD33^+^CD11b^+^HLA-DR^−^ MDSCs in patient with locally advanced rectal cancer undergoing neoadjuvant chemo-radiotherapy. We analyzed peripheral blood and tumor samples from 25 patients with T2 and T3 stages, who received nCRT, at two different time points, before and after nCRT. The results were then compared to 10 HDs. We demonstrated that in this relative early stage cancer, levels of peripheral MDSCs were not elevated, and could not predict response to nCRT. As opposed to circulating MDSCs, intratumoral MDSCs were elevated in cancerous tissue relative to their level in normal mucosa. Moreover, increased frequencies of intratumoral MDSCs with suppressive capabilities in patients before nCRT, correlated with non-responsiveness and minimal benefits by means of pathologic tumor response rate, when compared with patients exhibiting relatively low frequencies of this intratumoral cell populations.

In this study, we also analyzed MDSC frequencies in both peripheral blood and tumor samples. This is in clear contrast to many published studies, which evaluated MDSC frequencies solely in blood samples from cancer patients (21). We found that in contrast to peripheral blood, in which the level of CD33^+^CD11b^+^HLA-DR^−^ MDSCs did not increase in this relatively early stage of LARC, their levels within the tumor were significantly higher relative to the adjacent normal rectal mucosa. Specifically, significant higher levels of intratumoral MDSCs (3.9 ± 1.7 for entire EMCs population, *P* < 0.05) were detected as compared with rectal mucosa (0.8 ± 0.24). In peripheral blood however, the total EMCs population in HDs (1.57 ± 0.39, *P* > 0.05) was comparable to the level seen in LARC patients (3.38 ± 1.85), but not to the level seen in metastatic patients (12.89 ± 3.55, *P* < 0.05 for comparison with both HD and LARC groups). Functionally, these examined MDSCs inhibit CD3/CD28-dependent T-cell proliferation. These results are supported by a previous study demonstrating that the increase in peripheral blood MDSCs in colorectal cancer patients is closely correlated with clinical cancer stage and tumor metastasis, as well as an between tumor tissues relative to normal paraneoplastic tissue (32). These findings suggest that while MDSC accumulation within the tumor microenvironment is an early process, the rise of MDSCs in circulation is a relatively late phenomenon, which can be correlated with metastatic disease.

Our results did not demonstrate any changes in MDSC frequencies associated with gender, tumor location in the rectum or age in this group of patients with LARC.

Since intratumoral MDSCs levels were elevated in LARC patients, we explored the possible role of intratumoral MDSCs as predictive marker for response to nCRT. Although combined treatment with Capecitabine and radiation in the neoadjuvant setting has been shown to decrease relapse in patient with LARC (2), only a relative small proportion of patients benefits from this treatment with an objective pathologic complete response rate of about 10% (5). In our study, we found that while circulatory MDSC levels could not distinguish between pathologic responders and non-responders to nCRT, the analysis of tumor samples from patients with LARC before nCRT revealed significantly lower frequencies of intratumoral CD33^+^HLA-DR^−^CD16^−^CD11b^+^ MDSCs in patients responding to the treatment compared with non-responders. These results suggest that MDSCs in a biopsy material could be used as predictive biomarkers before nCRT to estimate likelihood to benefit from this therapy.

Other markers than MDSCs may predict differential response to nCRT. To address this, we analyzed somatic mutations in some of our patients. It is noteworthy, that K-ras mutations are more common in the non-responder group, and were not found in the responder group. Future study to correlate K-ras status with MDSCs level in the tumor is warranted.

Part of MDSCs effects are exerted by direct inhibition of tumor-infiltrating cytotoxic T-cells (33). However, the number of tumor-infiltrating T-cells in the analyzed samples did not correlate with rate of pathologic response to nCRT. This is in contrast to studies demonstrating that CRC tumors which display higher infiltrating cytotoxic lymphocyte correlate with a better prognosis, especially in the setting of mismatch repair instability tumors (34). It is plausible that while T cell infiltration is important for continuous tumor suppression, it is less critical in the response to nCRT, which directly affect MDSCs. In addition, in several studies of tumor-infiltrating lymphocytes in CRC (35, 36) T cell activity was not evaluated but only their numbers. Their activity can be affected by MDSC numbers. Overall, our results suggest that MDSCs may have a role in rectal cancer resistance to nCRT, an effect which is not limited only to T-cell inhibition.

It has been previously suggested that 5-Fluorouracil, and its derivative Capecitabine, which is the drug used in nCRT, bares direct anti-MDSC effect (37). In our study, we show that there is a robust increase in circulatory MDSC 6–8 weeks after nCRT, when patients were admitted for surgery. This effect was similar in responders and non-responders. In contrast, within the tumor, a significant decrease in MDSC content was observed, perhaps due to the effect of radiotherapy. It is reasonable that some of those MDSCs will eventually find their way into the tumor and will eventually contribute to disease recurrence and metastasis, supporting the role of post-therapy circulating MDSCs level (rather than the pre-treatment level) as prognostic marker.

In recent years, cancer immunotherapy using immune checkpoint inhibitors has led to dramatic and durable responses in patients with previously untreatable tumors. However, some tumor types, microsatellite stable colorectal cancers (MSS CRC) among others, failed to respond to this drug (38). Several mechanisms have been proposed to explain these results, such as the low expression of checkpoint molecules such as PD-1, PD-L1, CTLA-4, LAG-3, and IDO in MSS vs. microsatellite instable (MSI) CRC. Though CRC does not have a good response rate to PD-1 pathway immunotherapy, our results suggest that at least in a subset of rectal cancer patients, diminishing immunosuppressive targets may render the tumors particularly good candidates for checkpoint immunotherapy. In the current study, we demonstrate that MDSC may be such a target in rectal cancer patient amenable to nCRT. We proposed that in patients with high levels of intratumoral MDSC levels, it is better to target MDSCs before or during nCRT, in order to improve treatment outcome. In such case, it can be further considered immunotherapy approach as depleted MDSCs will improve T cell activity.

Although having the limitation of a small cohort, the strength of this work comes from its prospective design, with all patients analyzed for their MDSC content before treated. This form a sound base for the use of intratumoral MDSC content as predictive marker for response to nCRT, and calls for larger cohort studies.

To conclude, here we show, for the first time, that intratumoral, but not circulating CD33^+^HLA-DR^−^CD16^−^CD11b^+^ MDSCs bare suppressive capabilities and are associated with decreased rate of pathological response to nCRT in patients with locally advanced rectal cancer. Collectively, our data should lead to more judicious use of nCRT, and to the use of personalized and optimized treatment modalities in response to MDSC level in the tumor. These approaches and new possible biomarkers will enable prediction of better treatment outcome, together with improved quality of life.

## Materials and Methods

### Patients

Patients who were diagnosed with rectal cancer during colonoscopy at Rambam Health Care Campus or referred for trans-rectal ultrasound (TRUS) due to recent prior colonoscopic diagnosis of rectal cancer were recruited to this study. All patients had chest and abdominal CT to rule out distant metastasis and were classified as LARC based on TRUS findings. Patients were classified as T2 (tumor invades to muscularis propria) or T3 (tumor invades through muscularis propria), according to the common TNM classification. Tumor and normal adjacent tissue in addition to 3–5 ml of peripheral blood were obtained from patients who underwent colonoscopy at Rambam. While only tumor and blood were collected from patients recruited prior to TRUS. In addition, normal rectal mucosa and peripheral blood were collected from 10 healthy donors (HDs) and 6 patients with metastatic disease during or close to the time of colonoscopy. After recruitment, clinical parameters were obtained for each patient, including imaging data and medical history. Patients were excluded from the study, if they were found to have distant metastasis or previous exposure to chemotherapy or radiotherapy. Patients were recruited based on the approval of the Institutional Ethical Committee at Rambam health care campus. All patients were then referred for nCRT which included local radiation and 825 mg/m^2^ capecitabine b.i.d, followed by tumor resection on week 9 or 10. Clinical parameters were acquired from the patient's medical records at the Oncology department, Rambam Health Care Campus, Haifa, Israel. After the nCRT treatment period patients underwent surgery at Rambam Medical Center, and assessment of the response grade was based on pathologic analysis described elsewhere (39). A complete pathologic response (tumor response grade [TRG] 3) was defined as no viable cancer cells, single cells, or small groups of cancer cells. Moderate response (TRG 2) was defined as residual cancer outgrown by fibrosis. Minimal response (TRG 1) was defined as significant fibrosis outgrown by cancer, and no response (TRG 0) was defined by extensive residual cancer. Supplemental Table 1 shows a comparison between TRUS-based staging at diagnosis and pathology-based post-operative staging, for each individual patient. Patients with TRG 1 and 2 were considered as responders, while patients in TRG 3 status were considered as non-responders. See Figure 1 for the study model.

### Flow Cytometry Analysis

Blood samples were collected from patients at baseline. In addition, tumor tissues obtained from patients were prepared as single cell suspension as described below. All samples were immunostained with various antibodies to identify specific cell populations. Specifically, white blood cells were defined as CD45+. Of those, early myeloid cells were defined as CD33+HLA-DR-. Of these, further division was made based on the presence or absence of CD11b/CD16 surface markers. The antibody mix included anti HLA-DR-FITC, anti-CD33-PE, anti CD11b-APC, anti-CD45-APC-Cy7, anti-CD16-PerCP, anti-CD3-PE, and anti-CD8-FITC. All antibodies were purchased from BioLegend. For whole blood samples, after immunostaining, the samples underwent red blood cell lysis. All samples were analyzed by Amnis® imaging flow cytometer using IDEAS® software (Millipore Sigma).

### Single Cell Suspension of Tumor Tissue

Tumor tissue obtained by biopsy during rectoscopy or by surgical removal were transferred to 10 cm plate and cut into ≤5 mm pieces in sterile medium (RPMI 1640, 20% fetal calf serum, 2% Pen-Strep solution). Tumor pieces were dissociated into single cells using GentleMacs™ device (Miltenyi Biotec) in the presence of Collagenase I and Dispase II, followed by 40 min incubation in 37°C. cells were centrifuged and pellet was resuspended in red blood cell lysis buffer for 10 min. Washed cells were passed through 40 μM cell strained and washed with Phosphate-buffered saline, centrifuged and resuspended with flow stain buffer. Subsequently, cells were ready for immunostaining by flow cytometry.

### Carboxyfluorescein Diacetate Succinimidyl Ester (CFSE) Lymphocyte Proliferation Assay

Fresh isolated lymphocytes from peripheral blood of 5 healthy volunteers were resuspended in PRMI culture medium containing 5% heat-inactivated fetal calf serum (FCS), to a final concentration of 1 × 10^6^ cells/mL. Different MDSC subpopulations were sorted using FACSARIA II cell sorter (BD biosciences). Lymphocytes were incubated for 5 min with 5 μM CFSE final concentration, washed twice with PBS, and resuspended in full culture medium containing 10% FCS. Lymphocytes were than stimulated with anti-CD3/CD28 beads (Invitrogen) in the presence of 100,000 cells of the different MDSCs subpopulations. After 48 h, cells were analyzed for the quantity of CFSE by FACSCalibur (BD biosciences) and analyzed by FlowJo software.

### Statistical Analysis

Sample size calculations were based on 90% confidence interval, 0.5 standard deviation, and margin of error +/−5%. Those led to a needed sample size of about 90 responses. However, this number of patients is exceeding the capabilities of our and many other medical centers. Therefore, and being a proof-of-concept study, we collected data of one third of that number. Statistical analyses were performed using GraphPad Prism 7.03. Averaged values are presented as the mean ± SD. When comparing two groups, we determined statistical significance using the two-tailed Student's *t*-test after applying the appropriate normality test to verify normal distribution of the MDSC (see Supplemental Table 2 for normality test criteria for the CD16–/CD11b+ subpopulations). When more than two groups were investigated, we performed one-way ANOVA statistical test. Multivariable analysis was based on Cox proportional hazards regression analysis and was used to determine the independent effects of a prognostic factor. *P* < 0.05 were considered statistically significant.

## Data Availability Statement

The datasets generated for this study are available on request to the corresponding author.

## Ethics Statement

The studies involving human participants were reviewed and approved by Rambam Health Care Campus Institutional Review Board. The patients/participants provided their written informed consent to participate in this study.

## Author Contributions

EH, YS, YC, and EEH: conception and design. EH and EEH: development of methodology. WK, DD, AB, and YF: acquisition of data (provided tissue, managed patients, analyzed specimens, provided facilities, etc.). EH and AD: laboratory procedures. EH and EEH: analysis and interpretation of data (including statistical analysis). EH, YS, YC, and EEH: writing, review, and/or revision of the manuscript. EEH: study supervision. All authors contributed to the article and approved the submitted version.

## Conflict of Interest

The authors declare that the research was conducted in the absence of any commercial or financial relationships that could be construed as a potential conflict of interest.

## Supplementary Material

Supplemental Figure 1 **(A)** Details of excluded patients and justification for exclusion. **(B)** Representative 3D reconstruction of TRUS imaging, showing the rectal lumen (RL), the four different layers of the rectum (M, mucosa; SM, submucosa; Mu, muscularis; S, serosa), rectal mass involving the muscularis layer (T), and numerous enlarged lymph nodes (purple arrowheads). **(C)** The response to nCRT was evaluated by pathologic examination, and patients were devided accordingly to four different response grades: 0, no regression; 1, residual dominant tumor mass with pbvious fibrosis and vasculopathy; 2, dominant fibrosis with few residual tumor cells; 3, no tumor cells. **(D–F)** Percentages of intratumoral CD45+ (D), CD33+HLADR– EMCs **(E)**, and CD16–CD11b+ MDSCs **(F)** were evaluated in patients according to their age.

Supplemental Figure 2 **(A)** Prevalence of circulating CD16–/CD11b+ MDSCs in in the plasma of stage II vs. stage III LARC patients. **(B)** Prevalence of CD16–/CD11b+ MDSCs within tumor tissue of stage II vs. stage III LARC patients.

Supplemental Table 1 Individual comparisons between staging at diagnosis (based on TRUS) and post-operation staging (based on pathological findings).

Supplemental Table 2 Normality test criteria for tissue CD16–/CD11b+ in responder and non-responder subpopulations.

## Abbreviations

CFSE: CarboxyFluorescein Succinimidyl Ester
EMC: Early Myeloid Cells
5FU: 5Flouriuracil
FACS: Fluorescence Activated Cell Sorting
HD: Healthy Donors
LARC: Locally Advanced Rectal Cancer
MDSC: Myeloid Derived Suppressor Cells
MSS: MicroSatellite Instable
MSI: MicroSatellite Stable
nCRT: Neoadjuvant ChemoRadioTherapy
TRG: Tumor Response Grade.
