# Supplementary figures and images for: Intratumoral HLA-DR−/CD33+/CD11b+ Myeloid-Derived Suppressor Cells Predict Response to Neoadjuvant Chemoradiotherapy in Locally Advanced Rectal Cancer
Source: Front Oncol. 2020 Aug 12;10:1375. doi: 10.3389/fonc.2020.01375 (PMC7435035; doi:10.3389/fonc.2020.01375)

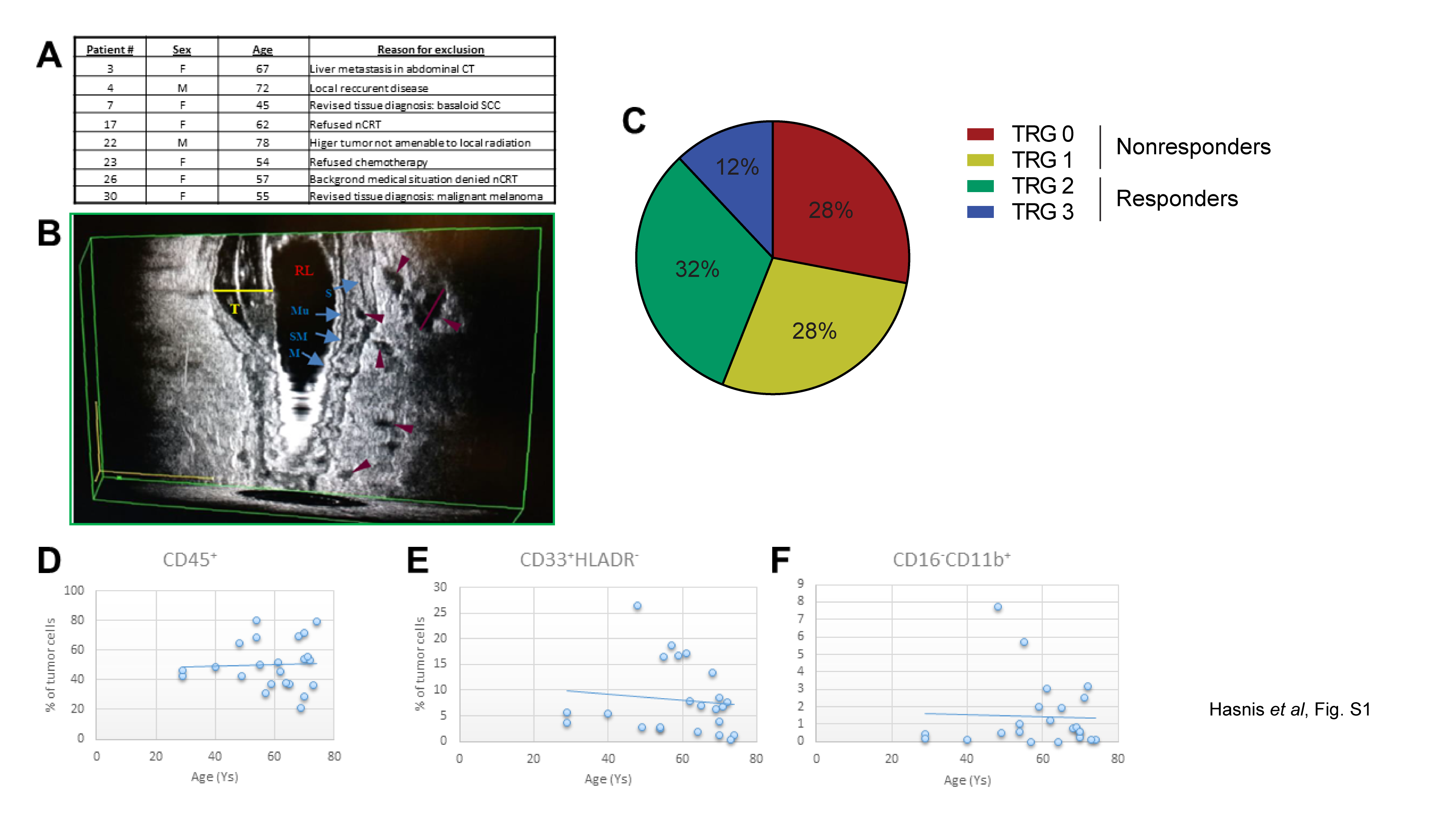

Supplement: Supplemental Figure 1 — (A) Details of excluded patients and justification for exclusion. (B) Representative 3D reconstruction of TRUS imaging, showing the rectal lumen (RL), the four different layers of the rectum (M, mucosa; SM, submucosa; Mu, muscularis; S, serosa), rectal mass involving the muscularis layer (T), and numerous enlarged lymph nodes (purple arrowheads). (C) The response to nCRT was evaluated by pathologic examination, and patients were devided accordingly to four different response grades: 0, no regression; 1, residual dominant tumor mass with pbvious fibrosis and vasculopathy; 2, dominant fibrosis with few residual tumor cells; 3, no tumor cells. (D–F) Percentages of intratumoral CD45+ (D), CD33+HLADR– EMCs (E), and CD16–CD11b+ MDSCs (F) were evaluated in patients according to their age. [file Image_1.TIF]

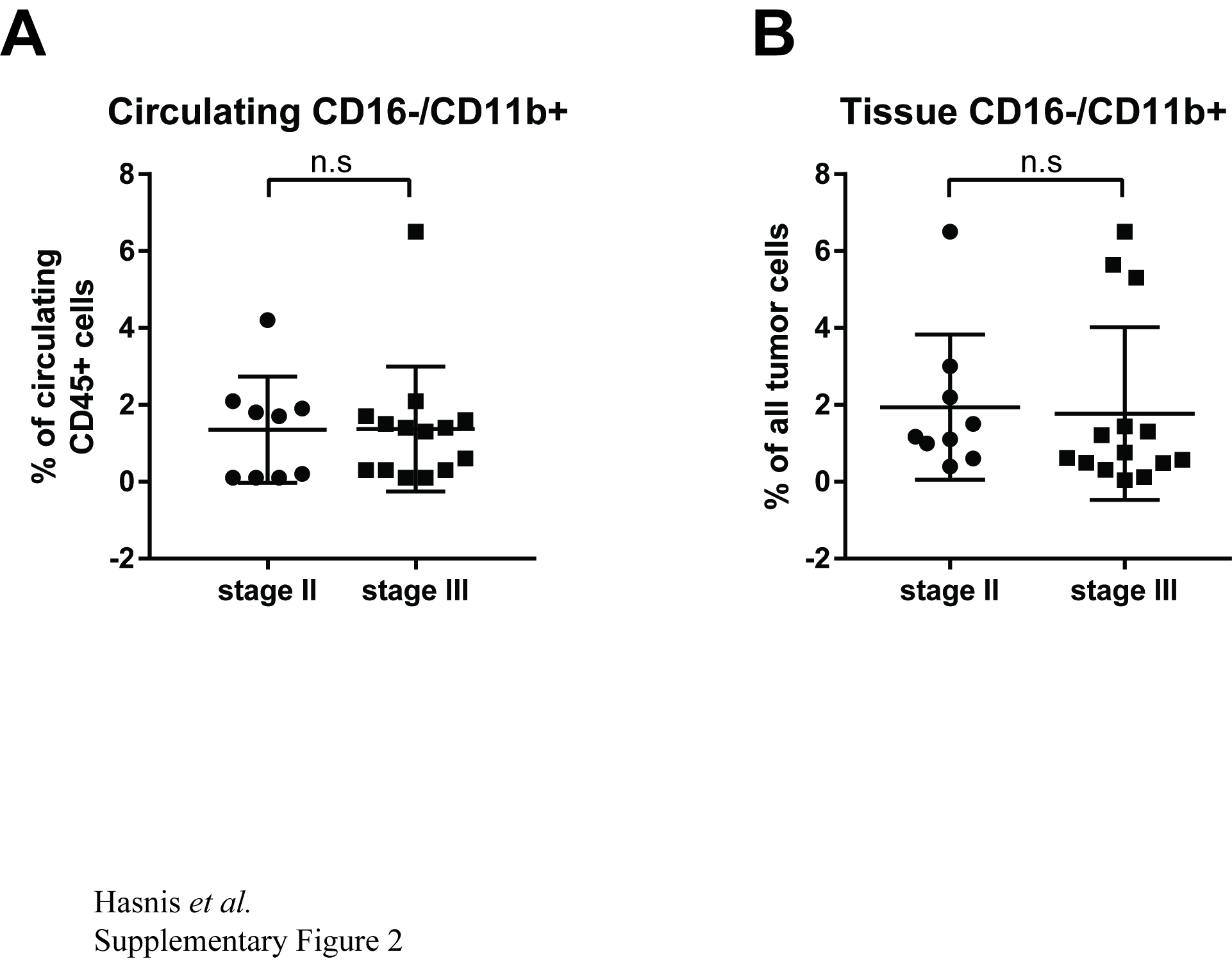

Supplement: Supplemental Figure 2 — (A) Prevalence of circulating CD16–/CD11b+ MDSCs in in the plasma of stage II vs. stage III LARC patients. (B) Prevalence of CD16–/CD11b+ MDSCs within tumor tissue of stage II vs. stage III LARC patients. [file Image_2.TIF]
