# Supplementary material for: Intratumoral HLA-DR−/CD33+/CD11b+ Myeloid-Derived Suppressor Cells Predict Response to Neoadjuvant Chemoradiotherapy in Locally Advanced Rectal Cancer
Source: Front Oncol. 2020 Aug 12;10:1375. doi: 10.3389/fonc.2020.01375 (PMC7435035; doi:10.3389/fonc.2020.01375)
Supplement: Supplemental Table 1 — Individual comparisons between staging at diagnosis (based on TRUS) and post-operation staging (based on pathological findings). [file Table_1.pdf]

Supplemental table 1: Individual comparisons between staging at diagnosis (based on TRUS) and post-operation staging (based on pathological findings)

Patient no.	Stage at diagnosis	Stage per post-op pathological findings
1	T3N1	T3N1
2	T3N0	Complete response
3	T3N1	T3N1
4	T2N0	T2N1
5	T3N0	Foci of ulcerating adenocarcinoma, 16 reactive lymph nodes
6	T3N1	T3N1
7	T3N1	T2N1
8	T3N1	T3N1
9	T3N0	Complete response
10	T3N1	T3N1
11	T3N1	T3N1
12	T3N1	T3N0
13	T3N0	T3N1
14	T3N1	T3N1
15	T3N1	T3N1
16	T3N1	T2N0
17	T2N1	T2N1
18	T3N1	Complete response
19	T3N0	T2N0
20	T2N0	T3N0
21	T2N1	T2N0
22	T3N1	T3N0
23	T3N1	T3N2
24	T3N1	Foci of ulcerating adenocarcinoma, 6 reactive lymph nodes
25	T3N0	T3N1
