# Supplementary material for: Intratumoral HLA-DR−/CD33+/CD11b+ Myeloid-Derived Suppressor Cells Predict Response to Neoadjuvant Chemoradiotherapy in Locally Advanced Rectal Cancer
Source: Front Oncol. 2020 Aug 12;10:1375. doi: 10.3389/fonc.2020.01375 (PMC7435035; doi:10.3389/fonc.2020.01375)
Supplement: Supplemental Table 2 — Normality test criteria for tissue CD16–/CD11b+ in responder and non-responder subpopulations. [file Table_2.pdf]

Supplemental table 2: Normality test criteria for tissue CD16-/CD11b+ in responder and nonresponder subpopulations

	Responders	Nonresponders
Number of values	11	14
Minimum	0.03489	0.196
25% Percentile	0.3088	0.5745
Median	0.5694	1.368
75% Percentile	0.7164	3.58
Maximum	1.101	6.5
Mean	0.5182	2.187
Std. Deviation	0.3001	2.118
Std. Error of Mean	0.09048	0.5661
Lower 95% CI of mean	0.3166	0.9642
Upper 95% CI of mean	0.7198	3.41
Sum	5.7	30.62
**D'Agostino & Pearson normality test**		
K2	0.3106	3.845
P value	0.8562	0.1462
Passed normality test (alpha=0.05)?	Yes	Yes
P value summary	ns	ns
